# Why not? Motivations for entering a volunteer register for clinical trials during the COVID-19 pandemic

**DOI:** 10.1007/s00228-022-03385-0

**Published:** 2022-09-14

**Authors:** Selena Russo, Marco Bani, Marco Terraneo, Valeria Quaglia, Giampaolo Nuvolati, Rebecca Cavaliere, Serena Capici, Marina Elena Cazzaniga, Maria Grazia Strepparava

**Affiliations:** 1 School of Medicine and Surgery, University of Milano-Bicocca, Milan, Italy; 2 Department of Sociology, University of Milano-Bicocca, Milan, Italy; 3 Royal College of Surgeons, University of Medicine and Health Sciences, Dublin, Ireland; 4 Phase 1 Research Centre, ASST Monza, Monza, Italy; 5 Clinical Psychology Unit, ASST Monza, Monza, Italy

**Keywords:** COVID-19, Vaccine, Clinical trial, Healthy volunteers, Motivations, Trust, Healthy volunteers register

## Abstract

**Backgrounds:**

Healthy volunteers play a key role in clinical trials and it is crucial to develop recruitment strategies that capitalise on their motivations and maximise their participation. The COVID-19 pandemic has shown the importance of finding motivated healthy volunteers for the development of new vaccines. Public registers represent a promising way to promote the participation of healthy volunteers in the research field, but their adoption is still limited. The current study aimed to explore the motivations of healthy volunteers to enrol in an Italian public register for clinical trials during the COVID-19 pandemic and their attitude toward participating in a phase 1 COVID-19 vaccine clinical trial. The impacts of different enrolling interview modalities (in person, by phone, by mail) on motivation, understanding of information and trust in researchers were also investigated.

**Methods:**

An online survey investigating experience with COVID-19, motivations to enrol, trust in researchers, political and healthcare authorities and pharmacological companies was presented to people applying as healthy volunteers in the public register for clinical trials at Phase 1 Unit Research Centre of ASST Monza, Italy, and considering to participate in a COVID-19 vaccine clinical trial. Data were collected in June 2021.

**Results:**

Altruistic motivations were the main driver for enrolling in the public register, while self-interested motivations were secondary. No gender differences were found. As for enrolling modalities, no differences emerged between in-person and interviews for motivation to enrol, understanding of information and trust in researchers. Email modality led to significantly lower volunteers’ satisfaction and understanding of information but similar trust in research.

**Conclusions:**

This study supports the validity of different interview modalities (in person and by phone) for the enrolment of healthy volunteers for clinical trials and highlights the positive role of public registers for the recruitment procedures.

**Supplementary Information:**

The online version contains supplementary material available at 10.1007/s00228-022-03385-0.

## Introduction

When considering human participants’ processes and motivations to volunteer for clinical studies, healthy volunteers present unique features that have made them the object of several empirical investigations [1, 2]. The pre-existing clinical conditions of patients entering clinical trials have been proven to be an important variable in shaping the motivational process underlying the enrolment process (i.e. being able to benefit from experimental treatments not yet available outside the clinical trial) [3]. Healthy volunteers do not derive any personal therapeutic benefits as they are not directly affected by the disease under study. In countries and contexts where they are licensed, monetary incentives to enrol are strong and debated motivating factors [4] often linked to the practice of serial participation and the over-representation of disadvantaged groups [5–7]. Other often reported motivating factors are more linked to feelings of personal gratification, and altruistic or moral rewards [8]. A systematic review examining healthy volunteers’ drivers to enrol in clinical studies and including 13 studies and more than 2000 healthy volunteers showed that financial rewards were the primary motivations to enrol [9]. Other motivations included contributing to science or the health of others, healthcare benefits, scientific interests and curiosity. In vaccine trials, healthy volunteers can be therefore motivated by self-interest and personal advantages such as protecting their health or access to free health benefits. Nevertheless, volunteering for vaccine clinical trials may be supported by robust prosocial attitudes, such as the desire to contribute to science and social health or to engage in an endeavour perceived as important for the whole community [2, 9]. Cattapan and colleagues [10] explored and compared motivations of healthy volunteers for a phase 1 trial of an Ebola virus vaccine and an adjuvant influenza vaccine trial and found a mix of altruistic and self-interested motivations.

Existing studies on the motivations of healthy volunteers in vaccine trials have involved particular populations and diseases, such as HIV/AIDS [11] or Ebola [10], but never an ongoing and severe health challenge such as the COVID-19 pandemic with a direct and vast impact on the global population.

The exceptional situation that the COVID-19 pandemic imposed provided a unique opportunity to advance our understanding of the motivational drivers underlying the decision to volunteer for vaccine development and testing. Recent studies reveal a willingness to participate in COVID-19 vaccine clinical trials ranging between 30 and 70%. An online survey conducted in France [12] on more than 3000 participants highlighted that while 75% of the respondents were willing to get the COVID-19 vaccine, only 48% of them would participate in a COVID-19 vaccine clinical trial. Older age, being male, being a healthcare worker and higher risk perception of infection were associated with willingness to participate in a COVID-19 vaccine clinical trial. In a convenience sample of Jordan adults, 36.1% of participants reported being willing to participate in vaccine clinical trials [13]. Major drivers were the desire to return to normal life and to help in finding a treatment. Among the reported barriers were not wanting to be challenged by the virus, fear of adverse outcomes, lack of time and mistrust in pharmaceutical companies. Out of 657 Uganda healthcare workers, between 70 and 42% were willing to participate in a hypothetical COVID-19 vaccine trial depending on vaccine trial requirements and concerns about the safety of the vaccine. Willingness to participate was associated with being male and lower educational level [14]. Abdelhafiz et al. [15] found that about 60% of 1500 Arab participants had positive attitudes toward participation in COVID-19 vaccine clinical trials. Willingness to participate was associated with a lower fear of the negative impact on health, greater knowledge of COVID-19 and greater trust in physicians and hospitals. Furthermore, a qualitative study exploring the experience of 31 participants in the Pfizer/BioNTech COVID-19 vaccine phase 3 clinical trial [16] revealed that participants’ reported motivations for trial participation can be relevant to overcome the concerns of others and could inform messaging and materials to promote COVID-19 vaccine programmes.

### Aims

This cross-sectional study aimed:

(i) to describe the motivational profiles of people who applied as healthy volunteers at the public register of the Phase 1 Unit Research Centre of ASST Monza (Italy) from which volunteers for phase 1 and phase 2 trials of the SARS-COVID-19 vaccine Covid-eVax (Takis and Rottapharm Biotech) had been chosen; and
(ii) to assess the impact of different enrolling interview modalities on information understanding, motivation to enrol, and trust in researchers in people considering enlisting in the public register for healthy volunteers of the Phase 1 Unit Research Centre of ASST Monza (Italy).

This study provides important data on motivations to volunteer in a healthy volunteer public register and in phase 1 COVID-19 vaccine trial in an Italian cohort of healthy volunteers furthering our understanding of the altruistic and self-interested triggers to volunteer for clinical trials.

## Methods

### Design, participants and procedure

A cross-sectional design was used. People that answered an open call for healthy volunteers to be enrolled in a register for clinical trials and contacted the Phase 1 Unit Research Centre of ASST Monza between June and September 2020 were invited to participate in the study. Although the call specifically referred to the possibility to be included in phase 1 clinical trial for a COVID-19 vaccine as well as future clinical trials, the possibility to enrol in the public register and to volunteer for the COVID-19 vaccine trial was advertised and presented jointly. Eligibility criteria for entering the healthy register and undergoing the interview to enrol for COVID-19 vaccine trials were (i) being 18 years old or older; (ii) being able to read and write Italian sufficiently to complete the survey; and (iii) being able to provide informed consent. There were no specific exclusion criteria. Prospective volunteers underwent an in-person interview, phone interview or email communication, depending on the possibility to arrange in-person interviews at the hospital study centre due to the COVID-19 restrictions and preventive measures. Ethical approval for the study has been received by the ASST Monza ethical committee (study n° 3692 Prot.0012887/21).

An invitation email describing the purposes of the study and a link to access the online survey was sent to 478 people who volunteered for enrolling in the clinical trial register. Electronic consent was obtained from all participants and participation was anonymous. The questionnaire took about 30 min to be completed. Data was collected in June 2021, nearly 1 year after the call for volunteers was advertised.

### Measures

Self-reported questionnaires elicited information on demographic characteristics, motivations, COVID-19-related information, and psycho-social dimensions.

- Six items on a 5-point Likert scale evaluated participants’ perception of physical, psycho-social and economic aspects of COVID-19. The items were combined by summing over respondents’ ratings to create the severity index, a score of the perception of the disease severity. This index has been proven to have sound psychometric properties [17].
- Two items investigated participants’ perception of the probability rated on a 4-point Likert scale (1—highly possible to 4—not at all possible) that someone would get infected in the country and in the participant’s town in the following weeks. Two items evaluated participants’ perception of the risk to die and having severe health issues due to COVID-19. Answers were given on Visual Analogue Scales ranging from 0 to 100.
- Four items investigated COVID-19-related worry. Two items rated on a 10-point rating scale investigated participants’ concerns of contracting COVID-19 and concerns about their friends/family members contracting the disease. Two items rated on a 5-point Likert scale asked about concern on the pandemic in the country and the region.
- COVID-19-related attitudes and beliefs were investigated with 5 items rated on a 5-point Likert scale addressing agreement with statements covering the severity of influenza and COVID-19 threat on human health, vaccines’ ability to control spread of infections and role of scientific investigations.
- Intensity of the motivation to enrol in the register for healthy volunteers and to enrol for the COVID-19 vaccine clinical trial was assessed with two items rated on a 10-point Likert scale. Furthermore, drivers to enrol in the register for healthy volunteers were assessed with 24 questions rated on a 5-point Likert scale classified as altruistic motivations, self-interested motivations or other motivations [10]. Data were organised in a 3-level variable (Strongly agree/agree; Neither agree/disagree; Disagree/strongly disagree).
- The 4-item Trust in Medical Researchers Generally—short form [18] was used to measure the level of trust of people in medical researchers.
- Six items on a 5-point Likert scale (1 = not at all to 5 = extremely reliable) explored participants’ trust in the national government, in the regional government, in the regional healthcare system, in the Hospital Centre, in the participant’s general practitioner and in healthcare professionals in managing the pandemic.
- Trust in pharmaceutical companies was explored using 4 items from the Edelman Trust Barometer 2020 [19]. Participants were asked to rate on an 11-point scale (top 5 boxes, positive; bottom 5 box, negative) their ethical perception of the industry on 4 dimensions, namely, purpose-driven, honest, vision and fairness.
- Participants’ health literacy was measured with the Italian version of the Single Item Literacy Screener (SILS). SILS is a single item designed to identify patients with limited reading ability who need help reading health-related materials [20, 21]. Response choices of SILS are 1—Never, 2—Rarely, 3—Sometimes, 4—Often and 5—Always. Cut-off > 2 is used to identify limited health literacy screening sensitivity. Scores greater than 2 were used to indicate inadequate health literacy.
- Participants’ satisfaction with enrol interview was explored with the Patient Satisfaction Questionnaire (PSQ) [22]. PSQ is a 5-item questionnaire measuring patient satisfaction by addressing their needs, their active involvement in the interaction, the quality of the information received, the emotional support received and the assessment of the global interaction. Answers were given on Visual Analogue Scales ranging from 0 to 100. An overall satisfaction score was obtained by averaging the responses to the five questions.
- One item explored participants’ perception of their understanding of the information received during the enrolment interview on a 10-point Likert scale (1—Not at all to 10—Extremely).
- One multiple-choice item asked participants to indicate the enrolling interview modality for the healthy volunteers at the Phase 1 Unit Research Centre of ASST Monza (Italy).

### Statistical analysis

Analyses included estimations of means, standard deviations and frequency distribution of the investigated variables. We contrasted participant groups using chi-square test, z-score test, unpaired *t* test, Mann–Whitney *U* test, and analysis of variance (ANOVA). Bivariate analyses investigated the association between the study variables. A set of chi-square tests of homogeneity compared frequency distributions of study participants’ motivations to volunteer with data from Cattapan et al. [10] on motivations to participate in Ebola and adjuvant influenza vaccine trials. All data analyses were performed using the statistical software package SPSS 27.0.

## Results

### Sample characteristics and COVID-19-related psycho-social variables

A total of 320 participants completed the questionnaires (response rate: 67%). Participants’ demographic characteristics are presented in Table 1. Study participants were predominantly male (61.9%) and had a mean age of 47.1 years. Data on participants’ experience, beliefs, worries, and severity perception of COVID-19 are reported in ESM Appendix 1.

**Table 1:** Sample demographic features (*N* = 320)

Demographics	*N* (%)	
**Age**	47.1 ± 12.6 (19–73)	
18–29	39 (12.2%)	
30–49	130 (40.6%)	
50–64	116 (36.2%)	
65 +	34 (10.6%)	
**Gender**		
Female	122 (38.1%)	
Male	198 (61.9%)	
**Region of residence**		
Lombardy region (where the study centre is located)	283 (88.4%)	
Other region	37 (11.6%)	
**Employment status**		
Employed (full-time, part-time or self-employed)	248 (77.5%)	
Not seeking (student, retired, home duties, not suitable for work)	50 (15.6%)	
Unemployed or actively seeking 1st employment	19 (5.9%)	
I prefer not to answer	3 (0.9%)	
**Education**		
** Low**		178 (55.6%)
Middle school (year 9 or below)	23 (7.2%)	
High school	155 (48.4%)	
** High**		142 (44.4%)
Bachelor degree	32 (10%)	
Master degree	86 (26.9%)	
Postgraduate degree	24 (7.5%)	
**Health literacy**—How often do you need to have someone help you when you read instructions, pamphlets or other written material from your doctor or pharmacy?		
** High health literacy**	167 (52.2%)	282 (88.1%)
1—Never		
2—Rarely	115 (35.9%)	
** Low health literacy**	33 (10.3%)	38 (11.9%)
3—Sometimes		
4—Often	5 (1.6%)	
5—Always	-	

Nearly half of the volunteers had family or friends infected by COVID-19 and more than 20% report the death of a relative or friends caused by COVID-19.

Trust variable data are reported in Table 2. Healthcare professionals and the study hospital were the most trusted entities followed by participants’ general practitioners, healthcare regional system and regional and national government.

**Table 2:** Trust in medical researchers, trust in institutions and trust in pharma companies (*N* = 320)

Trust measures	
**Trust in institutions** (1 to 5)	
Italian national government	2.87 ± 0.95
Regional government	2.58 ± 1.09
Healthcare regional system	2.9 ± 1.09
Healthcare professionals	4.36 ± 0.67
Study hospital	4.15 ± 0.71
General practitioner	3.75 ± 1.06
**Trust in pharma** (0 to 10)	
Purpose-driven	7.53 ± 1.82
Honest	6.65 ± 1.86
Vision	7.19 ± 1.82
Fairness	6.52 ± 2.14

### Motivational profile: triggers to enrol

Motivational dimensions and intensity of motivation to take part in the COVID-19 vaccine trial and to enrol in the healthy register for phase 1 clinical trials are described in Table 3. The intensity of motivation to enrol in the COVID-19 vaccine trial and the healthy register for phase 1 clinical trials were both very high and did not differ (*t*(638) = 0.562, *p* = 0.089). Altruistic motivations were the leading driver to enrol with the item “I wanted to contribute to the health of others” receiving the highest scores, followed by “I wanted to contribute to the advancement of science” and “I wanted to help society”.

**Table 3:** Participants’ motivations to enrol in the healthy register and in the COVID-19 vaccine trial (*N* = 320)

**Motivations to enrol**	Study sample (*N* = 320)
**Intensity of motivation to enrol in the COVID-19 vaccine trial** (1 to 10)	9.3 ± 1.15
**Intensity of motivation to enrol in the healthy register** (1 to 10)	9.25 ± 1.1
**Motivation to enrol in the healthy register—dimensions** (1 to 5)	
Altruistic motivations	4.34 ± 0.61
Self-interested	1.77 ± 0.71
Other motivations	2.28 ± 0.63

No gender difference emerged for intensity of motivation to enrol in the healthy register (*U* = 10,408, *z* = 0.906, *p* = 0.365; mean rank female = 151.28, mean rank male = 151.28) and in the COVID-19 vaccine clinical trial (*U* = 98,288, *z* =  − 0.025, *p* = 0.980; mean rank female = 145.86, mean rank male = 146.08). A set of *t* test explored gender differences in motivation dimensions. No gender differences emerged for altruistic (*M* = 0.026, 95% CI [− 0.115, 0.166], *t*(318) = 0.368, *p* = 0.713), self-interested motivations (*M* = 0.072, 95% CI [− 0.087, 0.232], *t*(318) = 0.892, *p* = 0.373) or other motivations score (*M* = 0.129, 95% CI [− 0.005, 0.264], *t*(295.76) = 1.9, *p* = 0.058). When considering participants’ personal experiences and involvement with COVID-19, no difference emerged for altruistic (*M* = 0.077, 95% CI [− 0.057, 0.212], *t*(318) = 1.128, *p* = 0.260), self-interested motivations (*M* =  − 0.025, 95% CI [− 0.181, 0.130], *t*(318) =  − 0.320, *p* = 0.749) or other motivations score (*M* = 0.119, 95% CI [− 0.018, 0.257], *t*(318) = 1.706, *p* = 0.089) between participants who had family members or friends hospitalised or sick because of COVID-19. Similarly, no difference emerged for altruistic (*M* =  − 0.025, 95% CI [− 0.184, 0.134], *t*(318) =  − 0.312, *p* = 0.755), self-interested motivations (*M* =  − 0.137, 95% CI [− 0.320, 0.046], *t*(318) =  − 1.474, *p* = 0.141) or other motivations score (*M* = 0.125, 95% CI [− 0.038, 0.287], *t*(318) = 1.511, *p* = 0.132) between participants who reported to have experienced a loss because of COVID-19 and those who did not.

A set of chi-square tests of homogeneity was run to compare motivation to enrol in our sample to data from Cattapan et al. [10] on motivation to enrol in an adjuvant influenza vaccine trial and an Ebola vaccine trial. Observed frequencies and percentages of participants’ answers for each study are reported in ESM Appendix 2.

When considering motivations to enrol in the Ebola vaccine trial scenario of Cattapan et al.’s study, probability distributions were not equal to those in our sample for two *altruistic* items, three *self-interested* items, and five items belonging to the *other motivations* category.

As for the *altruistic* drivers, a higher proportion of participants in our sample selected “Strongly agree/agree” and a lower proportion selected “Neither agree/disagree” for the item “I wanted to help my community”, while a statistically lower proportion of participants in our study selected “Strongly agree/agree” for the item “I wanted to participate in something important”. For the *self-interested* drivers “I wanted to receive an incentive”, “I wanted to receive reimbursement of my out-of-pocket expenses”, and “I knew that I would receive compensation for any injury resulting from the trial”, all pairwise comparisons were statistically significant with “Strongly disagree/disagree” higher in our sample and the other two options collecting lower proportions than in Cattapan et al.’s Ebola vaccine scenario. For the *other motivation* items, post hoc tests revealed that all pairwise comparisons were statistically significant for the item “I felt a duty to participate” with “Strongly agree/agree” higher in our sample. An opposite trend emerged for the items “I was curious about the study”, “I wanted to have a new experience/something to do” and “I saw media coverage of the issue/illness” where a statistically lower proportion of participants in our study selected “Strongly agree/agree”, while a higher proportion selected “Strongly disagree/disagree”. As for the item “I felt that others will view my participation positively”, a statistically higher proportion of participants in our study selected “Strongly disagree/disagree”.

When comparing our data with those of the adjuvant influenza vaccine trial scenario of Cattapan and colleagues, probability distributions were not equal for the altruistic drivers, three *self-interested* items and five items belonging to the *other motivations* category. As for the *altruistic* drivers “I wanted to help my community” and “I wanted to contribute to the health of others”, a lower proportion of participants in our sample selected “Strongly disagree/disagree” than in Cattapan et al.’s study. A higher proportion of participants in our sample selected “Strongly agree/agree” for the altruistic driver “I wanted to help to control this disease/infection”. A lower proportion of participants in our sample selected “Strongly agree/agree” while a higher proportion selected “Strongly disagree/disagree” for the two *self-interested* drivers “I wanted to receive reimbursement of my out-of-pocket expenses” and “I wanted to receive an incentive”. Differently, in the self-interested item “I wanted access to or time with medical professionals”, a higher proportion of participants in our study selected “Strongly agree/agree”, and a higher proportion selected “Strongly disagree/disagree” than in the adjuvant influenza vaccine scenario. As for the *other motivation* items, the post hoc tests highlighted that a higher proportion of participants in our study selected “Strongly agree/agree” for the items “I felt a duty to participate” and “I was curious about the study”, while for the items “I wanted to have a new experience/something to do” and “I was influenced by my friends/family”, a lower proportion of participants in our sample selected “Strongly agree/agree”, while a higher proportion selected “Strongly disagree/disagree”. A higher proportion of participants in our study selected “Strongly disagree/disagree” in the item “I have a connection to the issue/illness through a friend/family member”.

### Enrolment process variables and outcomes

Sources of information for entering the volunteer register and interview mode are described in Table 4. Data on participants’ satisfaction with the interview and understanding of information received during the consultation by interview modes are reported in Table 5.

**Table 4:** Information sources and enrolment process variables

Information sources and enrolment process variables	*N* = *320*
**Information sources**	
Social media	87 (27.2%)
Radio/television	58 (18.1%)
Word of mouth	51 (15.9%)
Institutional communication from the hospital study centre	53 (16.9%)
Institutional communication from universities, research centres or regional healthcare institutions	16 (5%)
Other	55 (17.2%)
**Interview mode**	
Face-to-face interview	131 (40.9%)
Phone interview	81 (25.3%)
Email questionnaire	74 (23.7%)
Not contacted yet	33 (10.3%)
Other	1 (0.3%)
**Decision to enrol**	
How sure are you about your decision to enrol? (1—Not at all to 10—Extremely)	8.97 ± 1.33

**Table 5:** Participant satisfaction with interview and information understanding by interview mode (*N* = 286)

	Face-to-face interview (*N* = 131)	Phone interview (*N* = 81)	Email questionnaire (*N* = 74)	TOT (*N* = 286)
**Participant satisfaction with the interview (PSQ—tot)** (1 to 100)	90.59 ± 11.75	86.5 ± 18.95	59.61 ± 31.59	81.42 ± 24.70
PSQ-1—needs addressed	91.18 ± 12.72	88.46 ± 18.82	59.57 ± 32.38	82.23 ± 24.96
PSQ-2—patient involvement	91.53 ± 12.62	85.67 ± 22.47	58.62 ± 32.71	81.35 ± 25.99
PSQ-3—adequacy of info	91.44 ± 14.29	88.88 ± 18.72	61.86 ± 33.13	83.06 ± 25.13
PSQ-4—emotional support	87.21 ± 18.98	81.72 ± 24.71	58.35 ± 33.56	78.19 ± 27.7
PSQ-5—overall interaction	91.56 ± 13.85	87.8 ± 19.059	59.64 ± 33.18	82.24 ± 25.66
**Information understanding** (1 to 10)	8.89 ± 1.23	8.46 ± 1.58	6.86 ± 2.67	8.20 ± 2.03
**Trust in researcher** (1 to 20)	15.22 ± 3.66	15.27 ± 3.57	15.96 ± 3.41	15.28 ± 3.71

A one-way ANCOVA was performed to explore whether participants’ perception of their understanding of information received at enrolling interview was different for the three interview modes. After adjustment for health literacy level, participants’ perception of information understanding was statistically significantly different between the interview modes, *F*(2, 282) = 31.598, *p* < 0.001, partial *η*^2^ = 0.183. Understanding increased from the email mode to the phone interview and face-to-face interview, in that order. Bonferroni post hoc analysis revealed that the increase in participants’ understanding from emails to face-to-face interviews (*M*_diff_ = 2.05, 95% CI [1.142, 2.67]) and to phone interviews (*M*_diff_ = 1.61, 95% CI [0.92, 2.31]) was statistically significant (*p* < 0.001). No statistically significant difference between face-to-face and phone interview mode was found.

A set of one-way Welch ANOVA was run to explore differences in participants’ satisfaction with the enrolling interview. The total score of participants’ satisfaction with the enrolling interview was statistically significantly different for the different interview modes, Welch’s *F*(2, 127.244) = 33.121, *p* < 0.001. As shown in Table 3, the total participants’ satisfaction score increased from the email questionnaire to the phone, and face-to-face interview mode, in that order. Games-Howell post hoc analysis revealed that the mean increase from email questionnaire to face-to-face interview (*M*_diff_ = 30.98, 95% CI [21.87, 40.07]) was statistically significant (*p* < 0.001), as well as the increase from email questionnaire to phone interview (*M*_diff_ = 26.91, 95% CI [16.86, 36.96], *p* < 0.001). No statistically significant difference in the total participant satisfaction score between face-to-face and phone interview modes emerged.

All participant satisfaction items showed the same pattern of results with email questionnaires presenting the lowest scores which are statistically different from both face-to-face and phone interview modes (Welch’s *F* between 32.282 and *p* < 0.001). No differences emerged between face-to-face and phone interview modes. Namely, participants perceived fewer needs addressed in emails when compared to face-to-face (*M*_diff_ =  − 31.62, 95% CI [− 40.981, − 22.25], *p* < 0.001) and phone interview mode (*M*_diff_ =  − 28.89, 95% CI [− 39.112, − 18.66], *p* < 0.001). Patient involvement was significantly higher in face-to-face (*M*_diff_ = 32.9, 95% CI [23.846, 42.235], *p* < 0.001) and phone interviews (*M*_diff_ = 27.04, 95% CI [16.26, 37.83], *p* < 0.001) than in email questionnaire mode. Information were reported as more adequate when received during face-to-face (*M*_diff_ = 29.58, 95% CI [19.93, 39.023], *p* < 0.001) and phone interviews (*M*_diff_ = 27.01, 95% CI [16.61, 37.041], *p* < 0.001) rather than via email. When contrasted with emails, face-to-face (*M*_diff_ = 28.86, 95% CI [18.78, 38.95], *p* < 0.001) and phone interviews (*M*_diff_ = 23.36, 95% CI [12.06, 34.67], *p* < 0.001) generated a statistically significant higher perception of emotional support in participants. The overall satisfaction with interaction with physician was statistically significantly lower in the email mode when compared with both face-to-face (*M*_diff_ =  − 31.93, 95% CI [− 41.57, − 22.29], *p* < 0.001) and phone (*M*_diff_ =  − 28.17, 95% CI [− 38.68, − 17.65], *p* < 0.001) interviews.

A one-way ANOVA was performed to explore whether participants’ trust in medical researchers was different in the three interview mode groups. No statistically significant differences were found, *F*(2, 283) = 1.116, *p* = 0.329.

## Discussion

To the best of our knowledge, this is the first study exploring motivations to enrol in a public national register of healthy volunteers for phase 1 clinical trials during the COVID-19 pandemic.

Participants in our study reported polarised beliefs about COVID-19 which was perceived as a serious threat to human health and a burden to the healthcare system. They furthermore reported positive attitudes toward vaccines and clinical trials considered primary means to contain the pandemic and improve the prevention and control of diseases.

Trust in public authorities was scattered with healthcare authorities (healthcare professionals, study hospitals and general practitioners) receiving higher levels of trust, while political authorities were perceived as less trustworthy. Our findings are not totally in line with data on Italian samples during the COVID-19 pandemic that highlights an unusually high level of trust in public authorities [23]. It needed to be noted, however, that they did not differentiate between political and healthcare institutions, limiting the opportunity to compare data and potentially explaining the difference. Our results on trust in public authorities suggest that considering the public’s level of trust in the agency promoting the enrolment in the register may be extremely relevant to design and tailoring enrolling campaigns and strategies as people may be more inclined to enrol when the agency promoting the undertaking is perceived as highly trustworthy.

The study mainly aimed to explore motivations to enrol in a public register of healthy volunteers and in a COVID-19 vaccine clinical trial. The motivational levels were very high for both and did not differ. A possible explanation may lie in the fact that enrolment in the public register was advertised jointly to the enrolment in the COVID-19 vaccine clinical trial and that enrolment in the public register was a precondition to access the vaccine trial. We furthermore must consider the national and historical context in which the study took place. When Italy, the first in the western countries, was reached by the COVID-19 pandemic, it was only possible to put in place very few measures to contain the clinical impact on individuals and the pressure on the healthcare system. They were dealing with the new disease all the world was talking about but no other western countries had to face yet. These factors along with an initial extreme level of uncertainty about the disease, its consequences, and its mechanisms may have created particular positive attitudes and beliefs about the COVID-19 vaccine as a means to contain and stop the pandemic. In this frame, the vaccine clinical trial presented in our study was conducted in the most affected region of the country. At present, there is no data to speculate whether and how these circumstances and factors had shaped motivations underlying the decision to enrol in the register in our sample. Furthermore, existing COVID-19-related literature on the topic reports on intention to participate in hypothetical COVID-19 trials rather than actual behaviours hampering the possibility to compare data as profound differences may lie between declared motives for a future intention and those for an intention that has been translated into a behaviour. Comparisons with data from international studies and further research addressing determinants of motivational drivers to enrol in healthy volunteer registers are needed. These data would help to understand whether people enrolling in healthy volunteers registers have more polarised or stronger motivational drivers than people enrolling for particular clinical trials and therefore informing whether and to what extent using national registers to recruit healthy volunteers for clinical trials may maximise participant retention rate.

As for the motivational drivers of participants in our study, results partially confirmed previous studies [9] in detecting that drivers to volunteer for healthy individuals included altruistic motivations. When compared to previous data on motivations of healthy volunteers to participate in clinical trials for Ebola and adjuvant influenza vaccines [10], in our sample, self-reported altruistic motivations played a stronger role while self-interested motivations were extremely less frequent. Although we need to caution about the particular effect that the COVID-19 pandemic may have had on study participants’ motivations, this result fosters discussion about possible differences in motivational profiles between volunteers enrolling in public vs private registers and whether and to what extend these differences play a role in reducing serial participation or early dropout rates which are often reported among US healthy volunteers [5, 24, 25]. Further data are needed.

No gender differences emerged in terms of self-interested, altruistic or other motivations category in our study, while previous studies reported females being more influenced by altruistic motivations than males [26, 27].

As for socio-demographic characteristics, our sample presented a higher percentage of individuals employed and with higher education in comparison with healthy volunteers from other countries such as the USA, China, Belgium and Singapore [5, 8, 25]. As lower socio-economic status has been proved to be linked to economic drivers and “serial participation”, high dropout rates, and over-representation of disadvantaged minorities [5–7] which pose ethical and safety concerns, some authors advocated for implementing and fostering the use of national registers to overcome these issues [6, 28] as they guarantee a higher control over participation in clinical trials and a reduction in time and costs related to the recruitment process [29–31].

Despite the advantages that national public registers to recruit and monitor healthy volunteers for clinical trials offer, their usage is limited. When looking at the Italian context where this study was run, only 11 registers for healthy volunteers exist and are active and only 3 of them are public (including the newly established ASST Monza Phase 1 Research Centre) [32]. In the country, only 3 studies on healthy volunteers have been conducted in 2019, two in 2018, one in 2017 and three in 2016 [33] confirming the difficulty to involve this particular type of population in phase 1 clinical studies. Reasons may be related to the scarcity of public registers of healthy volunteers, the length of the procedures to obtain the authorisation of clinical trials and also the limited awareness among the public opinion of the importance of pharmacological research [34]. A survey of nearly 500 stakeholders (including clinical research organisations, academic clinical trial units, and industry) casts Italy among the less desirable countries to select for clinical trials due to the burdensome bureaucratic procedures and ethics committees approval [35, 36].

The second study aim was to assess the impact of three enrolling interview modalities on information understanding, satisfaction with enrolling interviews, and trust in researchers in people considering enlisting in the healthy volunteer public register of the Phase 1 Unit Research Centre of ASST Monza (Italy) and volunteering for a COVID-19 vaccine clinical trial. The level of trust in researchers was generally high and there were no differences across the three interview modes. However, understanding of information and satisfaction with the enrolling interview were significantly lower for email communications while no differences emerged between in-person and phone interviews.

These findings support the possibility of enrolling healthy volunteers in a register using phone interviews, increasing the possible number of volunteers that can be involved, and minimising the logistic burden for participants as it often represents a barrier for recruitment [8]. As for the negative impact of the email communication modality on participants’ understanding of information and satisfaction with enrolling modalities in our study, it cautions against using this interview/communication mode in the context of enrolment of healthy volunteers. A previous randomised controlled trial [37] assessed the effectiveness of an electronic portal for the recruitment of healthy volunteers and found that the portal was more effective and required less effort and costs when compared with phone calls and letters. However, neither the understanding of information received nor the trust in researchers was assessed.

### Limitations

There are several limitations to our study. A first limitation is related to its cross-sectional design, as we are unable to assess the stability of drivers over time. It would have been particularly interesting to assess whether the lessening of the pandemic emergency would have led to a decline in the motivation to participate in future clinical trials. Furthermore, it was not possible to separate motivation to enrol in the register and motivations to enrol for the COVID-19 vaccine clinical trial. A second limitation is the lack of a comparison group preventing the opportunity to contrast healthy volunteers’ data with community comparisons who refused when offered. A third limitation is represented by the 1-year window between the decision to be enrolled in the public register and the completion of the survey when the pandemic situation was partially modified, hampering the opportunity to provide motivational profiles at the time of enrolment. Finally, our enrolling interview modalities did not include online/video interviews which we encourage to include and assess in future investigations.

### Conclusion

Healthy volunteers play a key role in the development of new vaccines and treatments and in the testing of existing ones, and it is crucial to use effective strategies to enrol new volunteers, maximising their level of understanding of information, satisfaction with the enrolling process and trust in researchers, and optimising the efforts for the recruitment procedures. This study supports the possibility to use different interview modalities for the enrolment of healthy volunteers in a public register for clinical trials.

## Supplementary Information

Below is the link to the electronic supplementary material.

Supplementary file1 (DOCX 18 KB)
