# Supplementary material for: Why not? Motivations for entering a volunteer register for clinical trials during the COVID-19 pandemic
Source: Eur J Clin Pharmacol. 2022 Sep 14;78(11):1791–800. doi: 10.1007/s00228-022-03385-0 (PMC9471028; doi:10.1007/s00228-022-03385-0)
Supplement: Supplementary file 1 — Supplementary file1 (DOCX 18 KB) [file 228_2022_3385_MOESM1_ESM.docx]

Appendix 1 – Participants’ psycho-social COVID19-related variables

| **PSYCHO-SOCIAL COVID19-RELATED VARIABLES** |  |
| --- | --- |
| **Experience with COVID-19** |  |
| Family or Friends with COVID19  Yes  No | 163 (50.9%)  157 (49.1%) |
| Family or Friends dead because of COVID19  Yes  No | 75 (23.4%)  245 (76.6%) |
| Assisting/assisted COVID19 patients  Yes  No | 17 (5.3%)  303 (94.7%) |
| Personal Negative Impact of Covid19  Yes  No | 103 (32.2%)  217 (67.8%) |
| **COVID19-related worry** |  |
| **Worried that I get infected**  (1-Not at all worried to 10- Extremely worried) | 4.94 ± 2.35 |
| **Worried that family and friends get infected**  (1-Not at all worried to 10- Extremely worried) | 3.91 ± 1 |
| **Worried about COVID19 emergency in Italy**  (1-Not at all worried to 5- Extremely worried) | 3.71 ± .75 |
| **Worried about COVID19 emergency in Lombardy**  (1-Not at all worried to 5- Extremely worried) | 3.88 ± .83 |
| **COVID19-perceived severity** |  |
| **Perception of COVID-19 Severity – Severity Index** (6 to 30) | 25.43 ± 2.88  (14-30) |
| **Severity Items:** (1=not at all severe to 5=very much severe) |  |
| Infection spread rate | 4.23 ± .72 |
| Symptoms | 3.87 ± .73 |
| Mortality risk | 3.75 ± .88 |
| Social impact | 4.29 ± .75 |
| Economic impact | 4.64 ± .59 |
| Impact on the healthcare system | 4.63 ± .60 |
| **Attitudes and beliefs about COVID-19**  (1=strongly agree to 5=strongly disagree) |  |
| **COVID-19 infection poses a serious threat to human health** | 1.4 ±.69 |
| Strongly agree/ agree | 302 (94.4%) |
| Neither agree/disagree | 13 (84.1%) |
| Disagree/strongly disagree | 5 (1.5%) |
| **Vaccination is the best possibility to control COVID-19 infection** | 1.29 ±.56 |
| Strongly agree/ agree | 310 (96.9%) |
| Neither agree/disagree | 8 (2.5%) |
| Disagree/strongly disagree | 2 (.6%) |
| **Influenza poses a serious threat to human health** | 2.4 ±.96 |
| Strongly agree/ agree | 180 (56.3%) |
| Neither agree/disagree | 97 (30.3%) |
| Disagree/strongly disagree | 43 (13.5%) |
| **Vaccination is the best method to control influenza infection** | 1.76 ±84 |
| Strongly agree/ agree | 258 (80.6%) |
| Neither agree/disagree | 54 (16.9%) |
| Disagree/strongly disagree | 8 (2.5%) |
| **Clinical trial research is essential to improve the prevention and control of disease** | 1.09 ±.39 |
| Strongly agree/ agree | 318 (99.4%) |
| Neither agree/disagree | - |
| Disagree/strongly disagree | 2 (0.6%) |
| **Covid19-related probabilities** |  |
| **Probability somebody gets COVID-19 in Italy – coming weeks**  (1-highly possible to 4-not at all possible) | 1.21 ± .42 |
| **Probability somebody gets COVID-19 in my city – coming weeks**  (1-highly possible to 4-not at all possible) | 1.29 ± .48 |
| **Probability health compromised because of COVID-19 – next 12 months** (0 to 100) | 28.51 ± 20.12 |
| **Probability to die of COVID-19 – next 12 months** (0 to 100) | 17.18 ± 20.05 |

Appendix 2 - Participants’ motivations to enrol

| **MOTIVATIONS TO ENROL** | | | |
| --- | --- | --- | --- |
|  | Study sample  N=320 | Adjuvant Influenza trial N=27 | Ebola vaccine trial N=22 |
| **Motivation to enrol in the healthy register – items** (1 to 5) | | | |
| **I felt a duty to participate (other)** | 4.58 ± .74 |  |  |
| Strongly agree/ agree | 298 (93.1%) | 16 (57.7%) * | 14 (63.6%)* |
| Neither agree/disagree | 15 (4.7%) | 9 (34.6%) | 5 (22.7%) |
| Disagree/strongly disagree | 7 (2.2%) | 2 (7.7%) | 3 (13.6%) |
| **I wanted access to or time with medical professionals (self-interested)** | 2.63 ± 1.22 |  |  |
| Strongly agree/ agree | 81 (25.3%) | 0* | 2 (9.1%) |
| Neither agree/disagree | 101 (31.6%) | 7 (26.9%) | 6 (27.3%) |
| Disagree/strongly disagree | 138 (43.1%) | 20 (73.1%) | 14 (63.6%) |
| **I felt an obligation to the person who requested my participation (other**) | 1.95 ± 1.14 |  |  |
| Strongly agree/ agree | 29 (9.1%) | 1 (3.8%) | 3 (13.6%) |
| Neither agree/disagree | 74 (23.1%) | 4 (15.4%) | 7 (31.8%) |
| Disagree/strongly disagree | 217 (67.8%) | 22 (80.8%) | 12 (54.5%) |
| **I wanted to be with a friend or family member who is participating (other**) | 1.52 ± .86 |  |  |
| Strongly agree/ agree | 7 (2.2%) | 1 (3.8%) | - |
| Neither agree/disagree | 43 (13.4%) | 3 (11.5%) | 6 (27.3%) |
| Disagree/strongly disagree | 270 (84.4%) | 23 (84.6%) | 16 (72.7%) |
| **I wanted to contribute to the advancement of science (altruistic)** | 4.58 ± .72 |  |  |
| Strongly agree/ agree | 299 (93.4%) | 22 (81.5%) | 20 (90.9%) |
| Neither agree/disagree | 15 (4.7%) | 4 (14.8%) | 1 (4.5%) |
| Disagree/strongly disagree | 6 (1.9%) | 1 (3.7%) | 1 (4.5%) |
| **I wanted to contribute to the health of others (altruistic)** | 4.63 ± .63 |  |  |
| Strongly agree/ agree | 302 (94.4%) | 24 (88.9%)* | 20 (90.9%) |
| Neither agree/disagree | 16 (5%) | 1 (3.7%) | 1 (4.5%) |
| Disagree/strongly disagree | 2 (0.6%) | 2 (7.4%) | 1 (4.5%) |
| **I was curious about the study (other)** | 3.05 ± .1.32 |  |  |
| Strongly agree/ agree | 134 (41.9%) | 21 (77.8%)* | 17 (76.2%)* |
| Neither agree/disagree | 83 (25.9%) | 3 (11.1%) | 4 (19%) |
| Disagree/strongly disagree | 103 (32.2%) | 3 (11.1%) | 1 (4.8%) |
| **I wanted to receive an incentive (e.g., money, tablet) (self-interested)** | 1.37 ± .82 |  |  |
| Strongly agree/ agree | 11 (3.4%) | 17 (63%)* | 13 (59.1%)* |
| Neither agree/disagree | 22 (6.9%) | 1 (3.7%) | 5 (22.7%) |
| Disagree/strongly disagree | 287 (89.7%) | 9 (33.3%) | 4 (18.2%) |
| **I wanted to participate in something important (altruistic)** | 3.66 ± 1.33 |  |  |
| Strongly agree/ agree | 197 (61.6%) | 22 (81.5%) | 20 (90.9%)* |
| Neither agree/disagree | 63 (19.7%) | 4 (14.8%) | 1 (4.5%) |
| Disagree/strongly disagree | 60 (18.8%) | 1 (3.7%) | 1 (4.5%) |
| **I wanted to have a new experience/something to do (other)** | 2.27 ± 1.33 |  |  |
| Strongly agree/ agree | 61 (19.1%) | 12 (44%)* | 12 (54.5%)* |
| Neither agree/disagree | 74 (23.1%) | 10 (36%) | 7 (31.8%) |
| Disagree/strongly disagree | 185 (57.8%) | 5 (20%) | 3 (13.6%) |
| **I was influenced by my friends/family (other)** | 1.33 ± .76 |  |  |
| Strongly agree/ agree | 8 (2.5%) | 1 (3.8 %)* | 2 (9.1%) |
| Neither agree/disagree | 21 (6.65) | 7 (26.9%) | 3 (13.6%) |
| Disagree/strongly disagree | 291 (90.9%) | 19 (69.2%) | 17 (77.3%) |
| **I knew that I would receive compensation for any injury resulting from the trial (self-interested)** | 1.34 ± .82 |  |  |
| Strongly agree/ agree | 11 (3.4%) | 1 (3.8%) | 5 (22.7%)* |
| Neither agree/disagree | 20 (6.3%) | 5 (19.2%) | 9 (40.9%) |
| Disagree/strongly disagree | 289 (90.3%) | 21 (76.9%) | 7 (36.4%) |
| **I saw media coverage of the issue/illness (other)** | 2.37 ± 1.32 |  |  |
| Strongly agree/ agree | 63 (19.7%) | 2 (8%) | 20 (90.9%)* |
| Neither agree/disagree | 85 (26.6%) | 7 (24%) | 1 (4.5%) |
| Disagree/strongly disagree | 172 (53.8%) | 18 (68%) | 1 (4.5%) |
| **I have a personal connection to the issue/illness (other)** | 1.84 ± 1.2 |  |  |
| Strongly agree/ agree | 36 (11.3%) | 5 (19.2%) | 3 (13.6%) |
| Neither agree/disagree | 45 (14.1%) | 6 (23.1%) | 4 (18.2%) |
| Disagree/strongly disagree | 239 (74.7%) | 16 (57.7%) | 15 (68.2%) |
| **I felt that others will view my participation positively (other)** | 1.98 ± 1.2 |  |  |
| Strongly agree/ agree | 41 (12.8%) | 4 (16%) | 5 (22.7%)* |
| Neither agree/disagree | 66 (20.6%) | 9 (32%) | 9 (40.9%) |
| Disagree/strongly disagree | 213 (66.6%) | 14 (52%) | 8 (36.4%) |
| **I wanted to participate in the development of a new vaccine (altruistic)** | 4.15 ± 1.06 |  |  |
| Strongly agree/ agree | 252 (78.8%) | 19 (70.4%) | 20 (90.9%) |
| Neither agree/disagree | 44 (13.8%) | 5 (18.5%) | 1 (4.5%) |
| Disagree/strongly disagree | 24 (7.5%) | 3 (11.1%) | 1 (4.5%) |
| **I wanted advance access to the vaccine (self-interested)** | 2.19 ± 1.27 |  |  |
| Strongly agree/ agree | 60 (18.8%) | 7 (26.9%) | 6 (27.3%) |
| Neither agree/disagree | 66 (20.6%) | 6 (23.1%) | 5 (22.7%) |
| Disagree/strongly disagree | 194 (60-6%) | 14 (50%) | 11 (50%) |
| **I wanted to help my community (altruistic)** | 4.4 ± .81 |  |  |
| Strongly agree/ agree | 281 (87.8%) | 21 (77.8%)* | 14 (63.6%)* |
| Neither agree/disagree | 32 (10%) | 3 (11.1%) | 7 (31.8%) |
| Disagree/strongly disagree | 7 (2.2%) | 3 (11.1%) | 1 (4.5%) |
| **I wanted to help society (altruistic)** | 4.46 ± .74 |  |  |
| Strongly agree/ agree | 290 (90.6%) | 21 (77.8%) | 18 (81.8%) |
| Neither agree/disagree | 24 (7.5%) | 4 (14.8%) | 3 (13.6%) |
| Disagree/strongly disagree | 6 (1.9%) | 2 (7.4%) | 1 (4.5%) |
| **I wanted to help to control this disease/infection (altruistic)** | 4.53 ± .75 |  |  |
| Strongly agree/ agree | 299 (93.4%) | 21 (76.9%)* | 20 (90.9%) |
| Neither agree/disagree | 13 (4.1%) | 3 (11.5%) | 1 (4.5%) |
| Disagree/strongly disagree | 8 (2.5%) | 3 (11.5%) | 1 (4.5%) |
| **I wanted to receive reimbursement of my out-of-pocket expenses (self-interested)** | 1.34 ± .82 |  |  |
| Strongly agree/ agree | 11 (3.4%) | 9 (34.6%)* | 5 (22.7%)* |
| Neither agree/disagree | 19 (5.9%) | 5 (19.2%) | 10 (45.5%) |
| Disagree/strongly disagree | 290 (90.6%) | 13 (46.2%) | 7 (31.8%) |
| **I wanted psychological benefits (feeling good about myself) (other)** | 2.48 ± 1.34 |  |  |
| Strongly agree/ agree | 81 (25.3%) | 10 (37%) | 8 (36.4%) |
| Neither agree/disagree | 74 (23.1%) | 9 (33.3%) | 6 (27.3%) |
| Disagree/strongly disagree | 165 (51.6%) | 8 (29.6%) | 8 (36.4%) |
| **I have a connection to the issue/illness through a friend/family member (other)** | 1.71 ± 1.1 |  |  |
| Strongly agree/ agree | 27 (8.4%) | 4 (15.4%)* | 1 (4.5%) |
| Neither agree/disagree | 43 (13.4%) | 8 (30.8%) | 2 (9.1%) |
| Disagree/strongly disagree | 250 (78.1%) | 15 (53.8%) | 19 (86.4%) |

Participants’ motivations to enrol in the healthy register and in the COVID-19 vaccine trial (N=320). Data from Cattapan et al. [6] on motivation to participate in adjuvant Influenza vaccine trial (N=27) and in Ebola vaccine trial (N=22). *Statistically significant difference at Chi-square tests of homogeneity p<.05; Post hoc analyses involved pairwise comparisons using multiple z-tests of two proportions with a Bonferroni correction. Statistical significance was accepted at the adjusted significant level p < .008.
